# Assessment of Nutritional Status, Health Parameters, Body Composition, and Their Predictors in Lebanese Taekwondo Athletes: A Cross-Sectional Study

**DOI:** 10.3390/sports13080264

**Published:** 2025-08-12

**Authors:** Maha Hoteit, Maroun Khattar, Jennifer Derassoyan, Yara Abou Khalil, Amal Haidar, Rana Baroud, Habib Zarifeh, Fadi Kibbeh, Nathalie Jbeily, Hassan Karaki, Nikolaos Tzenios, Zahra Sadek

**Affiliations:** 1 PHENOL Research Group (Public Health Nutrition Program-Lebanon), Faculty of Public Health, Lebanese University, Beirut 6573, Lebanon; marounkhattar95@outlook.com (M.K.); jennifer_derassoyan@hotmail.com (J.D.); yara.r.aboukhalil@gmail.com (Y.A.K.); amalhaidar3135@gmail.com (A.H.); ranabaroud.2000@gmail.com (R.B.); 2 Institut National de Santé Publique, d’Epidémiologie Clinique et de Toxicologie-Liban (INSPECT-LB), Beirut 1100, Lebanon; 3 Department of Primary Care and Population Health, University of Nicosia Medical School, Nicosia 24005, Cyprus; 4 Lebanese Taekwondo Federation, Sin el fil, Beirut 9099, Lebanon; dr.habibzarifeh@gmail.com; 5 Faculty of Education, University of Sciences and Arts in Lebanon, Beirut P.O. Box 13-5053, Lebanon; amirleban@gmail.com; 6 Faculty of Social and Political Sciences, University of Lausanne, CH-1015 Lausanne, Switzerland; nathaliejbeily@gmail.com; 7 Physical Therapy Department, Faculty of Public Health, Section I, Lebanese University, Beirut 6573, Lebanon; hkaraki@ul.edu.lb (H.K.); zahrasadek81@hotmail.com (Z.S.); 8 Faculty of Public Health, Charisma University, London EC1V 7QE, UK; 9 Physical Therapy Department, Faculty of Public Health, Islamic University of Lebanon, Khaldeh P.O. Box 30014, Lebanon

**Keywords:** athletic, health, Lebanon, nutritional deficiencies, optimal nutrition, sports performance

## Abstract

Background: Taekwondo (TKD) athletes’ nutritional and health statuses and body composition are critical to their physical performance and overall fitness. In Lebanon, TKD is widely practiced; however, there is a significant gap in the literature regarding the nutritional and health profiles of its athletes. This study aimed to assess the nutritional status, anemia prevalence, body composition, and other health-related characteristics, among Lebanese TKD athletes. Additionally, it explored the determinants of normal hemoglobin (Hb) levels, blood pressure, normal muscle mass, and normal fat mass. Methods: A cross-sectional study was conducted between January and July 2023, involving 110 TKD athletes. Hemoglobin and hematocrit levels were measured to assess anemia, while body composition was evaluated using a bioelectrical impedance analyzer. Blood pressure was also recorded. Household dietary diversity was assessed using the Food Consumption Score, and additional data on sociodemographic factors, training frequency, and supplement or medication use were gathered through a structured questionnaire. Logistic regression models were applied to identify predictors of normal Hb levels, hypertension, and optimal muscle and fat mass. Results: Results showed that male athletes had significantly higher rates of normal Hb (*p*-value = 0.013) and muscle mass percentages (*p*-value < 0.001), while females had higher rates of normal blood pressure (*p*-value = 0.002) and were more likely to use iron supplements (*p*-value = 0.002) and painkillers (*p*-value = 0.041). Normal fat mass was positively associated with normal Hb levels (aOR: 11.98, *p*-value = 0.033). Female gender was linked to a lower likelihood of normal muscle mass (aOR: 0.13, *p*-value < 0.001) and hypertension (aOR: 0.19, *p*-value = 0.009). Higher training duration (10 h or more per week) (aOR: 3.46, *p*-value = 0.04) and normal BMI (aOR: 4.93, *p*-value = 0.003) were positively associated with normal muscle mass. Normal BMI (aOR: 14.68, *p*-value < 0.001) was positively associated with normal fat mass. Conclusion: These findings underscore the importance of individualized dietary interventions to enhance athletes’ overall health and performance, through the optimization of athletes’ body composition, and the prevention of deficiencies, especially iron deficiency.

## 1. Introduction

Based on the ‘General Theory of Fighting Arts’ (GTFA), martial art is defined as systematic bodies of belief, knowledge, and practice related to techniques of attack and defense against human adversaries, incorporating both physical and non-physical aspects. The GTFA recognizes the diverse philosophical and cultural roots of martial arts, acknowledging their use for self-defense, mental wellness, and competition [1]. The ‘World Taekwondo’ (WT) is a form of martial arts characterized by its emphasis on powerful kicks, particularly head-height and spinning kicks, which are often awarded more points in sparring competitions, and global popularity [2,3]. It also highlights the philosophy of TKD, as practiced by Jae Hun Kim and others, which emphasizes character development alongside physical training, by focusing on cultivating virtues like courtesy, integrity, perseverance, self-control, and indomitable spirit, which aims to improve not only martial arts skills but also the overall well-being and character of the practitioner [2,3].

TKD is suggested to improve physical activity due to positively impacting muscle strength, muscular endurance, aerobic capacity, agility, speed, and flexibility [4]. When it comes to physical performance, optimal nutrient intake is a key component, as nutrition provides the energy needed for biological processes [5]. In fact, besides being important for overall health and well-being, proper nutrition is critical for athletes willing to reach their highest levels of performance [5,6]. Based on the “American College of Sports Medicine”, the consumption of adequate energy both in amount and timing is crucial for maximizing training outcomes and maintaining health [7]. For carbohydrates, recommendations range from 3 to 12 g per kilogram body weight (g/kg BW) depending on the type of training intensity or competition [7]. Recommendations for protein range from 1.2 to 2 g/kg BW to support recovery, remodeling, metabolic adaptation, and protein turnover, and sometimes a higher intake can be recommended for a short period of time to maintain fat-free mass (FFM) [7]. As for fat, intake should be maintained within 20–35 percent of total energy intake (TEI), which is important for providing energy and the absorption of fat-soluble vitamins [7]. As can be seen, physical fitness and training are greatly affected by an athlete’s nutritional status [5,6]. Given its high importance, assessing nutritional status of athletes is critical to avoid deficiencies that might negatively impact their performance. For instance, a study that assessed dietary intake among TKD players revealed inadequate intake of iron and calcium [8], both of which are of concern in athletes [7], highlighting the need for proper nutrition guidance [8]. Moreover, protein and fiber intakes were shown to be inadequate among TKD players in India [9], warranting close monitoring of dietary intake. Iron deficiency is shown to affect around 10% and 35% of male and female athletes, respectively [10]. With or without anemia, iron deficiency can weaken muscle function, and negatively impact physical and mental performance, warranting immediate medical intervention [7]. Plus, anemia, usually assessed by measuring hemoglobin (Hb) or hematocrit (Ht) levels, decreases maximal oxygen uptake and diminishes oxygen carrying capacity, which is the main cause of diminished exercise capacity [11,12]. This makes the assessment of Hb and Ht levels in athletes crucial. Other micronutrients of concern in athletes are calcium; vitamin D, whose deficiencies negatively impact bone health; and antioxidants, which protect cell membranes from oxidative damage caused by chronic training [7]. Other than nutritional status, body composition is a fundamental component used to reflect the health status of athletes and is essential for the optimization of athletic performance [13]. For instance, an appropriate physique is a determinant of success and health of athletes, as well as their performance [14]. A higher muscular strength enables a person to potentiate quickly and to a higher extent while decreasing injury risk and strongly contributes to an athlete’s overall performance [15]. Given its high level of importance, safeguarding the long-term health and performance of athletes should receive great attention, as physical stress in times of training and competitions can cause alterations to body composition and eventually can be harmful to athletes [7]. This can be done through careful monitoring of body composition parameters (FFM, fat mass (FM), bone health, and hydration status), knowing the optimal physique of an athlete, and staying away from potentially injurious actions that may negatively impact body composition [7]. While specific studies on TKD players in Lebanon are scarce, insights from broader research on martial arts provide knowledge on the potential effects of martial arts on health status as evidenced in systematic reviews [16]. Despite the valuable insights provided by studies done in different countries, there is a pressing need for studies targeting TKD players in Lebanon, given the popularity of this sport in the country. Having context-specific data is crucial to developing efficient interventions tailored to the needs of TKD players in Lebanon. As such, the current study was conducted in order to assess (1) the nutritional status, (2) the prevalence of low Hb and Ht levels, (3) body composition, and (4) other health-related characteristics (chronic diseases, supplements/medications use, etc.), as well as the determinants of normal hemoglobin levels, hypertension, normal muscle mass, and normal fat mass in Lebanese TKD players.

## 2. Methods

### 2.1. Study Design and Sampling

A cross-sectional study was conducted between January and July 2023 and involved 110 participants. A convenient sampling method was employed, initiated through outreach to the Lebanese TKD Federation and the national team via email to attract a diverse group of WT athletes. Those expressing interest gave implied verbal consent initially, followed by written consent before completing the questionnaire. Strict measures for confidentiality and privacy were assured, and participants were screened for eligibility before inclusion.

During the initial phase of the study, 161 individuals showed interest in participation. After assessing interested individuals for eligibility, 51 were excluded as they did not meet the following inclusion criteria: aged 8 to 64 years, being Lebanese nationals, holding a black belt in TKD (including both coaches and players of both genders), refrained from eating and drinking coffee or any caloric beverages for at least 2 h before the body composition analysis. Exclusion criteria involved non-compliance with fasting and those who refused to fill the questionnaire.

### 2.2. Data Collection

#### 2.2.1. Anemia

To assess anemia, Hb and Ht levels were assessed using the “Compo Lab TS” anemia test, together with needles, blood lancets, and a lancing device, and measurements were taken by trained dietitians. For Hb, cutoffs used to define anemia in participants were based on the World Health Organization’s guidelines [17] as follow: <130 g/L in males aged between 15 and 65 years old; <120 g/L in nonpregnant females aged between 15 and 65 years old, nonpregnant girls aged between 12 and 14 years old, and boys aged between 12 and 14 years old; <115 g/L in children aged 5–11 years. For Ht, normal ranges were considered between 40% and 54% in males, and between 36% and 48% in females [18].

#### 2.2.2. Anthropometric Measurements and Body Composition

Weight and height were measured using standardized procedures by the same trained investigator to minimize errors and guarantee precision and consistency in data collection. Participants were weighed with shoes and socks off using the i30 body composition analyzer, accurate to the nearest 0.1 kilograms (kg) [19]. The i30 body composition analyzer is a professional foldable body composition assessment tool; it also provided segmental analysis of body composition, FM, FFM, protein, minerals, skeletal muscle mass (SMM), and lean body mass (LBM) [19]. It has a high correlation with the gold standard technique—dual X-ray absorptiometry (DEXA), is of a high quality, easy to use, and user-friendly, with an on-screen scale imaging system (measured weight ranges: 2~250 kg (resolution: 50 g); measured height ranges: 60.0~220.0 cm) [19]. Height measurements were taken barefoot in a standing position using a portable stadiometer, accurate to the nearest 0.1 centimeters (cm). After collecting the weight and height of every participant, the body mass index (BMI) was computed by dividing weight (kg) by the height squared (m^2^). The WHO cut-off points were used to categorize participants based on their BMI, and BMI-for-age was used for school-children and adolescent participants [20,21]. Participants were thus classified as either having a healthy BMI (normal), or an unhealthy BMI (have underweight, overweight, or obesity).

#### 2.2.3. Hypertension

Blood pressure was measured using the “Omron” blood pressure monitor [22]. Hypertension was defined using the “European Society of Hypertension” (ESH) guidelines, which recommend a threshold of >140/90 millimeters of mercury (mmHg) for the diagnosis of hypertension [23].

#### 2.2.4. Nutritional Status

Household dietary diversity

The food consumption score (FCS) is a global tool reflecting the number of individual foods consumed over a reference period. It asks about the household’s frequency of consumption of eight different food groups over the seven days preceding the survey [24]. The tool also explores whether the reported consumption of a person is typical, and the number of meals eaten by the persons or their household the previous day [24]. In short, it assesses household dietary diversity (HDD). A high (≥42) and a low (<42) HDD were therefore defined by calculating the FCS based on the following formula [24]: (starches × 2) + (pulses × 3) + vegetables + fruit + (meat × 4) + (dairy products × 4) + (fats × 0.5) + (sugar × 0.5).

#### 2.2.5. Sociodemographic, Lifestyle, and Medical Characteristics Questionnaire

A questionnaire was used to collect the participants’ following information: age, gender, residency, marital status, educational level, employment status, role (coach, player, or both), number of workout hours per week, the presence of chronic diseases, smoking status, energy drinks and alcohol consumption, and medications or supplements use.

### 2.3. Data Management and Statistical Analysis

Data was managed and validated on Excel then transferred to the “Statistical Package for the Social Sciences, the IBM SPSS Statistics 21” for analysis. Descriptive statistics were used to summarize quantitative variables (frequencies, percentage, means, and standard deviations (SDs)). Comparative statistical Chi-square test was performed to check for associations between variables. Logistic regression models were performed to assess the determinants of the dependent variables (anemia, hypertension, BMI, muscle mass percentage, fat mass percentage) at a 95% confidence level. Variables entered in the various models were chosen based on the bivariate analyses, whose results are shown in Supplementary Materials.

### 2.4. Ethical Considerations

The study protocol was approved by the Ethical Committee of the Lebanese University (#217/6 July 2022), adhering to the Declaration of Helsinki principles [25]. Participants provided written informed consent, with a focus on confidentiality, privacy, and the voluntary nature of participation with the right to withdraw at any time. Consent forms were obtained from participants and their parents if their age was below 18 years, with a focus on confidentiality and privacy, and that participation was entirely optional and that they had the right to withdraw at any time. All data was collected and maintained with strict confidentiality and privacy.

### 2.5. Consent for Publication

Written informed consent for publication of this manuscript and any accompanying data, tables, or figures has been obtained from all study participants (or their legal guardians). A copy of the written consent is available for review by the editor of this journal upon request.

## 3. Results

### 3.1. Sociodemographics, Lifestyle, and Medications and Supplements Use

The sociodemographic characteristics, lifestyle, and diet quality of participants are shown in Table 1. About two-third were males (67.3%). Almost 75% were adults, with the mean age ± SD (years) of the overall study population being 24.3 ± 10.5. The majority were residing in Beirut and Mount Lebanon (76.4%). Almost half of the participants were both a TKD coach and a TKD player. The majority reported training less than 10 h per week (65.5%). Energy drinks and alcohol consumption were reported by 29.1% and 40% of participants, respectively. The prevalence of energy drink consumption was significantly higher among males compared to females (*p*-value = 0.014). Table 2 shows medications and supplements use by the study participants. Almost 17% reported taking painkillers, 15% reported taking anti-inflammatory medications, and 20% reported taking multivitamin supplements. Magnesium supplements were the most reported by the study participants (22.7%), followed by vitamin C supplements (18.2%). Almost all the participants had an acceptable HDD, reflecting a good nutritional status. The prevalences of painkiller medication use (*p*-value = 0.041) and iron supplement use (*p*-value = 0.002) were significantly higher among females compared to males.

### 3.2. Medical Characteristics, Anthropometry, and Body Composition of the Study Participants

Table 3 summarizes the medical characteristics and body composition characteristics of the study participants. Overall, 8.2% of participants reported having at least one chronic disease. About one-third had high blood pressure (HBP), and 15.5% had low hemoglobin levels, indicating the presence of anemia. The majority of participants had a healthy BMI and normal muscle mass percentage; however, almost 57% had fat mass percentage out of the normal range. Based on gender, the prevalence of high blood pressure was significantly higher among males compared to females (*p*-value = 0.002); the prevalence of low Hb levels was significantly higher among females compared to males (*p*-value = 0.013); and the prevalence normal muscle mass percentage was significantly higher among males compared to females (*p*-value < 0.001).

### 3.3. Factors Associated with Normal Hemoglobin Levels, High Blood Pressure, Normal Muscle Mass Percentage, and Normal Fat Mass Percentage

Supplementary Table S1 shows the variables significantly correlated with normal Hb levels in our study sample. These variables were entered in the binary logistics model. The determinants resulting from the regression are shown in Table 4. Having a normal fat mass percentage increased the likelihood of having normal Hb levels (aOR: 11.98, *p*-value = 0.033).

Supplementary Table S2 shows the variables significantly correlated with HBP in our study sample. These variables were entered in the binary logistics model. The determinants resulting from the regression are shown in Table 4. Being a female decreased the likelihood of HBP (aOR: 0.19, *p*-value = 0.009).

Supplementary Table S3 shows the variables significantly correlated with normal muscle mass percentage in our study sample. These variables were entered in the binary logistics model. The determinants resulting from the regression are shown in Table 4. Being a female decreased the likelihood of having normal muscle mass percentage (aOR: 0.13, *p*-value < 0.001). Participants who train 10 h or more weekly were 3.46 times more likely to have normal muscle mass percentage compared with those who train less than 10 h weekly (aOR: 3.46, *p*-value = 0.04). Participants with a healthy BMI were 4.93 times more likely to have normal muscle mass percentage compared with those who have an unhealthy BMI (aOR: 4.93, *p*-value = 0.003).

Supplementary Table S4 shows the variables significantly correlated with normal fat mass percentage in our study sample. These variables were entered in the binary logistics model. The determinants resulting from the regression are shown in Table 4. Participants with a healthy BMI were 14.68 times more likely to have a normal fat mass percentage compared with those having an unhealthy BMI (aOR: 14.68, *p*-value < 0.001).

## 4. Discussion

To our knowledge, this study is the first to assess and evaluate the body composition parameters, anemia status, hypertension status, dietary behaviors, and dietary supplements use in Lebanese TKD athletes. The findings thus provide valuable insights, as the assessed variables are considered crucial components that affect performance. Based on our findings, the majority of TKD players were shown to have a normal BMI and normal muscle mass percentage; however, the majority were shown to have a fat mass percentage out of the normal range. Maintaining normal fat mass percentage is crucial when it comes to performance, which makes monitoring the body composition of TKD players, especially fat mass percentage, essential. For instance, muscle power performance and sport-specific anaerobic performance were shown to be negatively correlated with fat mass percentage in TKD athletes [26,27]. In our study, participants with a healthy BMI were more likely to have normal fat and muscle mass percentages compared with participants having an unhealthy BMI; therefore, in addition to regularly monitoring body composition parameters, we suggest working closely with TKD players to maintain a healthy BMI through proper nutrition and training routines. Our findings also show that participants who train 10 h or more weekly are more likely to have normal muscle mass percentage, highlighting the importance of training frequency when it comes to optimizing body composition parameters and eventually performance. Our results revealed that TKD players with normal fat mass percentage were more likely to have normal Hb levels compared with those having fat mass percentage out of the normal range, further highlighting the importance of maintaining a healthy BMI and body composition parameters in TKD players. In fact, anemia likelihood was shown to be higher among people with excess body fat [28], and having obesity may disrupt the homeostasis of iron in the body, resulting in anemia [29]. Our results also showed that about one-third of participants have HBP, with females being less likely to have it compared with males. This can be due to many factors, such as NSAID use, as about 15% of participants reported taking anti-inflammatory medications. In addition, some participants reported taking anti-hypertensive medications, implying that they already have HBP. However, HBP is not associated with training hours, thus with TKD training in our study. In contrast, undergoing TKD training regularly was shown to be protective against HBP and its related health risks [30], highlighting the important role TKD training plays in regulating HBP. Since hypertension diagnosis needs monitoring of blood pressure over an extended period of time, and not just a onetime measurement, further studies targeting blood pressure in TKD players over extended periods are needed to get better insights into the effect of TKD training on blood pressure. Taken together, the current findings highlight the importance of following a balanced diet in enhancing the performance and overall health of athletes. Given the significant prevalence of low Hb (15.5%) and Ht (23.6%) in our sample, implying the presence of anemia among 15.5% to 23.6% of the participants, dietary modifications, particularly focusing on iron-rich foods and optimizing iron absorption, can significantly improve participants’ iron status and potentially enhance their performance [31,32]. Furthermore, given the high prevalence of abnormal fat mass percentage in our sample (57.3%), individualized dietary intervention to improve body composition and optimize energy levels for training and competition is crucial [33]. This can be achieved by creating a calorie deficit combined with increased physical activity, especially for those who train for less than 10 h weekly. However, doing this without the help of professionals is challenging for athletes. For instance, a study done in Jordan revealed that while many TKD athletes possess adequate nutritional knowledge, they often struggle to translate this knowledge into practical dietary choices, which suggests a gap between knowledge and application, necessitating interventions aimed at improving dietary habits [34]. This highlights the importance of having professional intervention from dietitians who provide the best source of dietary information [34]. Moving forward, it is recommended that future studies examine short- and long-term interventions that can effectively monitor the dietary habits of TKD players and evaluate their physical health and performance by conducting food frequency questionnaires (FFQs) and 24 h recalls, and tests that give insights on their performance. In addition, due to the scarcity of literature in this area, more studies globally and regionally should investigate the impact of nutrition on the performance and wellbeing of TKD athletes in their respective populations, to provide a comprehensive understanding of the role proper nutrition plays in optimizing health and performance, particularly considering the increasing prevalence of hypertension, obesity, and anemia worldwide.

### Strengths and Limitations

Our study has several strengths that contribute to the understanding of the health of TKD athletes in Lebanon. This study pioneers as being the first to assess the nutrition status, body composition, dietary behavior, anemia, and hypertension in the Lebanese TKD athletes’ population. To ensure the validity of our results, strict exclusion criteria were adopted, minimizing confounding effects and strengthening the internal validity of our findings. In addition, our findings have practical implications, as the valuable data provided can be utilized to initiate targeted nutrition programs and to conduct more research targeting TKD athletes. Despite these strengths, our study has some limitations worthy of mention. Our sample size was limited and might not be representative of the Lebanese population of TKD players. Plus, one-time measurements of SBP and DBP are not sufficient for diagnosing hypertension but should be recorded over a period of time. Moreover, our study mostly relied on self-reported data, which are subject to recall bias and potential inaccuracies. Future research should aim to explore the nutritional and health statuses of TKD players using a larger sample size representative of the Lebanese TKD population, and monitoring blood pressure over a longer period of time to be able to diagnose hypertension in TKD practitioners as it has negative impacts on their overall health. Moreover, assessing dietary intake in terms of energy and macro- and micro-nutrients might give better insights into this population’s dietary patterns and nutritional status.

## 5. Conclusions

In conclusion, this study highlights the importance of proper nutrition in optimizing body composition parameters and performance in athletes. In addition, it sheds light on the importance of monitoring anemia, blood pressure, and medication and supplement use in athletes, as they impact their health and performance. We recommend that every professional athlete should be assessed regularly for proper nutrition, training routine, and health indicators (anemia, hypertension, etc.). This requires a professional team, including dietitians, physiotherapists, and medical doctors, and is crucial to optimizing athletic performance.

The study protocol was approved by the Ethical Committee of the Lebanese University (#217/6 July 2022), adhering to the Declaration of Helsinki principles. Participants provided written informed consent, with a focus on confidentiality, privacy, and the voluntary nature of participation with the right to withdraw at any time. Consent forms were obtained from participants or their parents if their age was below 18 years, with a focus on confidentiality, privacy, that participation was entirely optional, and that they had the right to withdraw at any time. All data were collected and maintained with strict confidentiality and privacy.

## Figures and Tables

**Table 1:** Sociodemographic characteristics, lifestyle, and diet quality of participants.

Variable		Overall (N = 110)		Male (N = 74)		Female (N = 36)		*p*-Value
		N	%	N	%	N	%	
Age Category	Children or adolescent	27	24.50%	17	23%	10	27.80%	0.583
	Adult	83	75.50%	57	77%	26	72.20%	
Governorate	Beirut and Mount Lebanon	84	76.40%	54	73%	30	83.20%	0.176
	North Lebanon and Akkar	7	6.40%	5	6.80%	2	5.60%	
	South Lebanon and Nabatiyeh	3	2.70%	1	1.30%	2	5.60%	
	Bekaa and Baalbek-Hermel	16	14.50%	14	18.90%	2	5.60%	
Marital Status	Not Married	19	17.30%	14	18.90%	5	13.90%	0.513
	Married	91	82.70%	60	81.10%	31	86.10%	
Education Status	University Level	66	60.00%	44	59.50%	22	61.10%	0.868
	School Level	44	40.00%	30	40.50%	14	38.90%	
Employment Status	Unemployed	55	50.00%	33	44.60%	22	61.10%	0.104
	Employed	55	50.00%	41	55.40%	14	38.90%	
Role	Both	56	50.90%	37	50.00%	19	52.80%	0.017
	Coach	25	22.70%	22	29.70%	3	8.30%	
	Player	29	26.40%	15	20.30%	14	38.90%	
Training Hours (Per Week)	<10 h	72	65.50%	46	62.20%	26	72.20%	0.298
	≥10 h	38	34.50%	28	37.80%	10	27.80%	
Energy Drinks	No	78	70.90%	47	63.50%	31	86.10%	0.014
	Yes	32	29.10%	27	36.50%	5	13.90%	
Alcohol	No	66	60.00%	41	55.40%	25	69.40%	0.158
	Yes	44	40.00%	33	44.60%	11	30.60%	
Smoking	No	95	86.40%	63	85.10%	32	88.90%	0.59
	Yes	15	13.60%	11	14.90%	4	11.10%	
HDD	Acceptable	109	99.10%	73	98.60%	36	100.00%	0.484
	Borderline	1	0.90%	1	1.40%	0	0.00%	

*p*-value < 0.05 indicates significant results. Abbreviations: HDD Household Dietary Diversity.

**Table 2:** Medication and supplement use among the study participants.

Variable		Overall (N = 110)		Male (N = 74)		Female (N = 36)		*p*-Value
		N	%	N	%	N	%	
Medications Use								
Vasodilator	No	109	99.10%	74	100.0%	35	97.2%	0.150
	Yes	1	0.90%	0	0.0%	1	2.8%	
Anticoagulants	No	109	99.10%	74	100.0%	35	97.2%	0.150
	Yes	1	0.90%	0	0.0%	1	2.8%	
Antiepileptics	No	107	97.30%	71	95.9%	36	100.0%	0.221
	Yes	3	2.70%	3	4.1%	0	0.0%	
Antidepressive	No	109	99.10%	74	100.0%	35	97.2%	0.150
	Yes	1	0.90%	0	0.0%	1	2.8%	
Painkillers	No	91	82.70%	65	87.8%	26	72.2%	0.041
	Yes	19	17.30%	9	12.2%	10	27.8%	
Blood Pressure	No	108	98.20%	72	97.3%	36	100.0%	0.320
	Yes	2	1.80%	2	2.7%	0	0.0%	
Anti-inflammatory	No	93	84.50%	63	85.1%	30	83.3%	0.806
	Yes	17	15.50%	11	14.9%	6	16.7%	
Anti-histamine	No	102	92.70%	71	95.9%	31	86.1%	0.062
	Yes	8	7.30%	3	4.1%	5	13.9%	
Supplements Use								
Multivitamin	No	88	80.00%	60	81.1%	28	77.8%	0.684
	Yes	22	20.00%	14	18.9%	8	22.2%	
Vitamin D	No	94	85.50%	66	89.2%	28	77.8%	0.111
	Yes	16	14.50%	8	10.8%	8	22.2%	
Vitamin C	No	90	81.80%	58	78.4%	32	88.9%	0.180
	Yes	20	18.20%	16	21.6%	4	11.1%	
Zinc	No	104	94.50%	70	94.6%	34	94.4%	0.974
	Yes	6	5.50%	4	5.4%	2	5.6%	
Magnesium	No	85	77.30%	59	79.7%	26	72.2%	0.378
	Yes	25	22.70%	15	20.3%	10	27.8%	
Iron	No	103	93.60%	73	98.6%	30	83.3%	0.002
	Yes	7	6.40%	1	1.4%	6	16.7%	
Calcium	No	105	95.50%	70	94.6%	35	97.2%	0.535
	Yes	5	4.50%	4	5.4%	1	2.8%	
Folic Acid	No	109	99.10%	74	100.0%	35	97.2%	0.150
	Yes	1	0.90%	0	0.0%	1	2.8%	
Vitamin B12	No	107	97.30%	73	98.6%	34	94.4%	0.204
	Yes	3	2.70%	1	1.4%	2	5.6%	
Vitamin A	No	110	100.00%	74	100.0%	36	100.0%	*-*
	Yes	0	0.00%	0	0.0%	0	0.0%	

*p*-value < 0.05 indicates significant results.

**Table 3:** Medical characteristics, anthropometry, and body composition.

Variable		Overall (N = 110)		Male (N = 74)		Female (N = 36)		*p*-Value
		N	%	N	%	N	%	
Presence of Chronic Diseases	Yes	9	8.20%	4	5.4%	5	13.9%	0.128
	No	101	91.80%	70	94.6%	31	86.1%	
Blood Pressure	Normal	73	66.40%	42	56.8%	31	86.1%	0.002
	High	37	33.60%	32	43.2%	5	13.9%	
Hemoglobin Level	Low	17	15.50%	7	9.5%	10	27.8%	0.013
	Normal	93	84.50%	67	90.5%	26	72.2%	
Hematocrit Level	Low	26	23.60%	14	18.9%	12	33.3%	0.095
	Normal	84	76.40%	60	81.1%	24	66.7%	
Body Mass Index	Unhealthy	38	34.50%	28	37.8%	10	27.8%	0.298
	Healthy	72	65.50%	46	62.2%	26	72.2%	
Muscle Mass%	Not Normal	30	27.30%	12	16.2%	18	50.0%	<0.001
	Normal	80	72.70%	62	83.8%	18	50.0%	
Fat Mass%	Not Normal	63	57.30%	42	56.8%	21	58.3%	0.875
	Normal	47	42.70%	32	43.2%	15	41.7%	

*p*-value < 0.05 indicates significant results.

**Table 4:** Predictors of normal hemoglobin, high blood pressure, normal muscle mass, and normal fat mass.

**Model 1**		**Normal Hemoglobin Levels**			
		**aOR**	**95% CI**		***p*-Value**
			**Lower**	**Upper**	
Gender					
Male (Reference)		-	-	-	-
Female		0.313	0.083	1.17	0.085
Muscle Mass Percentage					
Not Normal (Reference)		-	-	-	-
Normal		1.78	0.43	7.34	0.425
Fat Mass Percentage					
Not Normal (Reference)		-	-	-	-
Normal		11.98	1.22	117.22	0.033
**Model 2**		**HBP**			
		**aOR**	**95% CI**		***p*-Value**
			**Lower**	**Upper**	
Age Category					
Children or Adolescent (Reference)		-	-	-	-
Adult		1.9	0.28	12.85	0.51
Gender					
Male (Reference)		-	-	-	-
Female		0.19	0.053	0.65	0.009
Fat Mass Percentage					
Not Normal (Reference)		-	-	-	-
Normal		0.38	0.11	1.3	0.12
Employment Status					
Unemployed (Reference)		-	-	-	-
Employed		1.27	0.41	3.88	0.67
Training Hours (week)					
<10 h (Reference)		-	-	-	-
≥10 h		0.52	0.15	1.73	0.28
BMI					
Unhealthy (Reference)		-	-	-	-
Healthy		1.07	0.32	3.51	0.91
Role					
Both (Reference)		-	-	-	-
Coach		1.63	0.48	5.54	0.43
Player		0.91	0.13	6.3	0.92
**Model 3**		**Normal Muscle Mass Percentage**			
		**aOR**	**95% CI**		***p*-Value**
			**Lower**	**Upper**	
Gender					
Male (Reference)		-	-	-	-
Female		0.13	0.045	0.38	<0.001
Training Hours (week)					
<10 h (Reference)		-	-	-	-
≥10 h		3.46	1.06	11.28	0.04
BMI					
Unhealthy (Reference)		-	-	-	-
Healthy		4.93	1.69	14.38	0.003
**Model 4**		**Normal Fat Mass Percentage**			
		**aOR**	**95% CI**		***p*-Value**
			**Lower**	**Upper**	
Age Category					
Children or Adolescent (Reference)		-	-	-	-
Adult		1.01	0.13	7.9	0.99
Education					
Illiterate to School (Reference)		-	-	-	-
University		0.33	0.07	1.42	0.14
Employment Status					
Unemployed (Reference)		-	-	-	-
Employed		0.79	0.25	2.44	0.68
Smoking Status					
No (Reference)		-	-	-	-
Yes		0.44	0.06	3.16	0.41
Training Hours (week)					
<10 h (Reference)		-	-	-	-
≥10 h		2.96	0.88	9.89	0.078
BMI					
Unhealthy (Reference)		-	-	-	-
Healthy		14.68	3.58	60.13	<0.001
Role					
Both (Reference)		-	-	-	-
Coach		0.71	0.17	2.9	0.64
Player		0.74	0.13	4.32	0.73

*p*-value < 0.05 indicates significant results. Abbreviations: aOR: adjusted odds ratio, BMI: body mass index, CI: confidence interval, HBP: high blood pressure. Variables entered in model 1: gender, muscle mass percentage, fat mass percentage. Variables entered in model 2: age, gender, fat mass percentage, employment status, training hours, BMI, role. Variables entered in model 3: gender, training hours, BMI. Variables entered in model 4: age, education, smoking status, employment status, training hours, BMI, role.

## Data Availability

The datasets generated and/or analyzed during the current study are available from the corresponding author on reasonable request.

## Supplementary Materials

The following supporting information can be downloaded at: https://www.mdpi.com/article/10.3390/sports13080264/s1, Table S1: Factors correlated with hemoglobin level; Table S2: Factors correlated with blood pressure; Table S3: Factors correlated with muscle mass percentage; Table S4: Factors correlated with fat mass percentage.

## Abbreviations

BMI	Body mass index
BW	Body weight
cm	Centimeters
DEXA	Dual X-ray absorptiometry
ESH	European Society of Hypertension
FCS	Food consumption score
FFM	Fat-free mass
FFQ	Food frequency questionnaire
FM	Fat mass
g	Grams
GTFA	General Theory of Fighting Arts
HDD	Household dietary diversity
Kg	Kilograms
LBM	Lean body mass
mmHg	Millimeters mercury
SD	Standard deviation
SMM	Skeletal muscle mass
TKD	Taekwondo
WHO	World Health Organization
WT	World Taekwondo
