# Supplementary material for: Assessment of Nutritional Status, Health Parameters, Body Composition, and Their Predictors in Lebanese Taekwondo Athletes: A Cross-Sectional Study
Source: Sports (Basel). 2025 Aug 12;13(8):264. doi: 10.3390/sports13080264 (PMC12390590; doi:10.3390/sports13080264)
Supplement: Supplementary file 1 [file sports-13-00264-s001.zip › sports-3765126-supplementary.pdf]

Table S1. Factors correlated with hemoglobin level

		Hemoglobin Level				P-value
		Deficient		Normal		
		N	%	N	%	
**Gender**	Male	7	41.2%	67	72.0%	**0.013**
	Female	10	58.8%	26	28.0%	
**Age Category**	Under 18 years old	2	11.8%	25	26.9%	0.183
	18 years old and above	15	88.2%	68	73.1%	
**Muscle Mass %**	Not Normal	11	64.7%	19	20.4%	**<0.001**
	Normal	6	35.3%	74	79.6%	
**Fat Mass %**	Not Normal	16	94.1%	47	50.5%	**0.001**
	Normal	1	5.9%	46	49.5%	
**Education Status**	University Level	13	76.5%	53	57.0%	0.132
	School Level	4	23.5%	40	43.0%	
**Employment Status**	Unemployed	7	41.2%	48	51.6%	0.429
	Employed	10	58.8%	45	48.4%	
**Presence of Chronic Diseases**	No	14	82.4%	87	93.5%	0.121
	Yes	3	17.6%	6	6.5%	
**Role**	Both	11	64.7%	45	48.4%	0.458
	Coach	3	17.6%	22	23.7%	
	Player	3	17.6%	26	28.0%	
**Training Hours Per Week**	Less than 10 hours per week	14	82.4%	58	62.4%	0.111
	10 hours or more per week	3	17.6%	35	37.6%	
**Smoking**	No	13	76.5%	82	88.2%	0.196
	Yes	4	23.5%	11	11.8%	
**Energy Drinks**	No	13	76.5%	65	69.9%	0.583
	Yes	4	23.5%	28	30.1%	
**Alcohol**	No	10	58.8%	56	60.2%	0.914
	Yes	7	41.2%	37	39.8%	
**BMI**	Unhealthy BMI	6	35.3%	32	34.4%	0.944
	Healthy BMI	11	64.7%	61	65.6%	

Table S2. Factors correlated with blood pressure

		Blood Pressure				P-value
		Normal		High		
		N	%	N	%	
**Gender**	Male	42	57.5%	32	86.5%	**0.002**
	Female	31	42.5%	5	13.5%	
**Age Category**	Under 18 years old	24	32.9%	3	8.1%	**0.004**
	18 years and above	49	67.1%	34	91.9%	
**Hemoglobin Level**	Deficient	14	19.2%	3	8.1%	0.129
	Normal	59	80.8%	34	91.9%	
**Hematocrit Level**	Deficient	20	27.4%	6	16.2%	0.192
	Normal	53	72.6%	31	83.8%	
**Muscle Mass %**	Not Normal	18	24.7%	12	32.4%	0.387
	Normal	55	75.3%	25	67.6%	
**Fat Mass %**	Not Normal	34	46.6%	29	78.4%	**0.001**
	Normal	39	53.4%	8	21.6%	
**Education Status**	University Level	42	57.5%	24	64.9%	0.458
	School Level	31	42.5%	13	35.1%	
**Employment Status**	Unemployed	44	60.3%	11	29.7%	**0.002**
	Employed	29	39.7%	26	70.3%	
**Presence of Chronic Diseases**	No	69	94.5%	32	86.5%	0.146
	Yes	4	5.5%	5	13.5%	
**Role**	Both	38	52.1%	18	48.6%	**<0.001**
	Coach	9	12.3%	16	43.2%	
	Player	26	35.6%	3	8.1%	
**Training Hours Per Week**	Less than 10 hours per week	43	58.9%	29	78.4%	**0.042**
	10 hours or more per week	30	41.1%	8	21.6%	
**Smoking**	No	65	89.0%	30	81.1%	0.250
	Yes	8	11.0%	7	18.9%	
**Energy Drinks**	No	53	72.6%	25	67.6%	0.583
	Yes	20	27.4%	12	32.4%	

**Alcohol**	No	48	65.8%	18	48.6%	0.084
	Yes	25	34.2%	19	51.4%	
**BMI (Healthy vs. unhealthy)**	Unhealthy BMI	19	26.0%	19	51.4%	**0.008**
	Healthy BMI	54	74.0%	18	48.6%	

Table S3. Factors correlated with muscle mass percentage

		Muscle Mass %				P-value
		Not Normal		Normal		
		N	%	N	%	
**Gender**	Male	12	40.0%	62	77.5%	**<0.001**
	Female	18	60.0%	18	22.5%	
**Age Category**	Under 18 years old	4	13.3%	23	28.8%	0.094
	18 years old and above	26	86.7%	57	71.2%	
**Education Status**	University Level	21	70.0%	45	56.2%	0.190
	School Level	9	30.0%	35	43.8%	
**Employment Status**	Unemployed	13	43.3%	42	52.5%	0.392
	Employed	17	56.7%	38	47.5%	
**Presence of Chronic Diseases**	No	26	86.7%	75	93.8%	0.227
	Yes	4	13.3%	5	6.2%	
**Role**	Both	13	43.3%	43	53.8%	0.266
	Coach	10	33.3%	15	18.8%	
	Player	7	23.3%	22	27.5%	
**Training Hours Per Week**	Less than 10 hours per week	25	83.3%	47	58.8%	**0.016**
	10 hours or more per week	5	16.7%	33	41.2%	
**Smoking**	No	23	76.7%	72	90.0%	0.070
	Yes	7	23.3%	8	10.0%	
**Energy Drinks**	No	24	80.0%	54	67.5%	0.199
	Yes	6	20.0%	26	32.5%	
**Alcohol**	No	20	66.7%	46	57.5%	0.382
	Yes	10	33.3%	34	42.5%	
**BMI**	Unhealthy BMI	16	53.3%	22	27.5%	**0.011**
	Healthy BMI	14	46.7%	58	72.5%	

Table S4. Factors correlated with fat mass percentage

		Fat Mass %				P-value
		Not Normal		Normal		
		N	%	N	%	
**Gender**	Male	42	66.7%	32	68.1%	0.875
	Female	21	33.3%	15	31.9%	
**Age Category**	Under 18 years old	7	11.1%	20	42.6%	**<0.001**
	18 years old and above	56	88.9%	27	57.4%	
**Education Status**	University Level	47	74.6%	19	40.4%	**<0.001**
	School Level	16	25.4%	28	59.6%	
**Employment Status**	Unemployed	22	34.9%	33	70.2%	**<0.001**
	Employed	41	65.1%	14	29.8%	
**Presence of Chronic Diseases**	No	57	90.5%	44	93.6%	0.552
	Yes	6	9.5%	3	6.4%	
**Role**	Both	33	52.4%	23	48.9%	**<0.001**
	Coach	21	33.3%	4	8.5%	
	Player	9	14.3%	20	42.6%	
**Training Hours Per Week**	Less than 10 hours per week	48	76.2%	24	51.1%	**0.006**
	10 hours or more per week	15	23.8%	23	48.9%	
**Smoking**	No	50	79.4%	45	95.7%	**0.013**
	Yes	13	20.6%	2	4.3%	
**Energy Drinks**	No	43	68.3%	35	74.5%	0.478
	Yes	20	31.7%	12	25.5%	
**Alcohol**	No	34	54.0%	32	68.1%	0.135
	Yes	29	46.0%	15	31.9%	
**BMI**	Unhealthy BMI	35	55.6%	3	6.4%	**<0.001**
	Healthy BMI	28	44.4%	44	93.6%	
